# Effects of Childhood Trauma on Remembering the Past and Imagining the Future in Schizophrenia

**DOI:** 10.1002/pchj.70044

**Published:** 2025-07-31

**Authors:** Yu‐qi Yang, Jia‐li Liu, Tao Chen, Han Wang, Ji‐fang Cui, Hai‐song Shi, Tian‐xiao Yang, Ya Wang, Gui‐fang Chen

**Affiliations:** ^1^ School of Psychology Capital Normal University Beijing China; ^2^ College of Elementary Education Capital Normal University Beijing China; ^3^ Affiliated Hospital Zunyi Medical University Zunyi China; ^4^ Brain and Mind Centre The University of Sydney Sydney Australia; ^5^ School of Psychology The University of Sydney Sydney Australia; ^6^ Institute of Education Statistics and Data Analysis China National Academy of Educational Sciences Beijing China; ^7^ North China Electric Power University Beijing China; ^8^ Neuropsychology and Applied Cognitive Neuroscience Laboratory State Key Laboratory of Cognitive Science and Mental Health, Institute of Psychology Beijing China; ^9^ Department of Psychology University of Chinese Academy of Sciences Beijing China

**Keywords:** childhood trauma, imagining the future, remembering the past, schizophrenia

## Abstract

Schizophrenia exhibits impairments in remembering the past (autobiographical memory, AM) and imagining the future (episodic future thinking, EFT). Childhood trauma is also associated with deficits in AM and EFT. However, it is not clear whether childhood trauma is associated with severer deficits in AM and EFT in schizophrenia. The present study aimed to examine the effect of childhood trauma on AM and EFT in schizophrenia. We recruited 41 schizophrenia patients with childhood trauma (SCZ + CT), 19 schizophrenia patients without childhood trauma (SCZ − CT), and 40 healthy controls (HC) to participate in this study. Participants underwent the autobiographical interview task, in which they were required to remember or imagine the most important events that occurred or would occur at different times and describe them. Results showed that SCZ + CT exhibited fewer internal details, and lower specificity, time/place richness, and thought/emotion richness in both AM and EFT compared with HC. Meanwhile, SCZ − CT showed lower time/place richness and thought/emotion richness in AM and EFT than HC. However, no significant difference was found between the two patient groups. In addition, AM showed more internal details and stronger phenomenological characteristics (e.g., specificity, time/place richness, etc.) than EFT, while EFT was more positive and important than AM in all participants. Both SCZ + CT and SCZ − CT groups exhibited AM and EFT impairments, and the SCZ + CT group had wider impairments than the SCZ − CT group compared with HC, although the direct comparison between SCZ + CT and SCZ − CT did not show significant differences. These results suggest that childhood trauma had a subtle effect on AM and EFT impairments in schizophrenia patients.

## 1 Introduction

Schizophrenia is a severe mental disease, and patients with schizophrenia usually suffer from a wide range of cognitive impairments (Cai et al. 2024; Liu et al. 2023; Styła and Zajenkowski 2023), including remembering the past (autobiographical memory, AM) and imagining the future (episodic future thinking, EFT) (Hallford et al. 2018; Berna et al. 2016). AM and EFT have many important functions in daily lives, such as promoting social bonding, improving self‐continuity, problem‐solving, farsighted decision‐making, and emotion regulation (Schacter et al. 2017; Sow et al. 2022). Deficits in remembering and imagining specific events are related to social dysfunction and clinical symptoms in schizophrenia patients (D'Argembeau and Raffard 2008; Herold et al. 2022; Huddy et al. 2016; Mehl et al. 2010).

Schizophrenia exhibited deficits in AM and EFT, including reduced specificity (a specific event means an event occurs at a specific place and time and lasts < 1 day, specificity means proportion of specific events), decreased richness of details (vivid and specific details about the event, e.g., time and place, perception, emotion/thought, etc.), and fewer positive events (Allé et al. 2020; Barry et al. 2019; Chen et al. 2016; Raffard et al. 2010; Wang et al. 2017; Yang et al. 2018). A meta‐analysis including 20 studies revealed that schizophrenia spectrum disorders exhibited a significant reduction in specificity (*g* = −0.97) and detail richness (*g* = −1.40) in AM, suggesting that schizophrenia is associated with an impaired capacity to retrieve unique memories from their past experiences, extract details, and reconstruct scenes in the mind (Berna et al. 2016). Another meta‐analysis including six studies in schizophrenia on EFT revealed that these patients were associated with reduced specificity and a number of details in EFT (*g* = −1.00) (Hallford et al. 2018).

Childhood trauma refers to a broad spectrum of adverse experiences such as physical, emotional, and sexual abuse, and physical and emotional neglect that occur during the formative years of a person's life and exceed one's ability to cope (Borrelli et al. 2024; Musicaro et al. 2019; Thomas et al. 2021). Research indicates childhood trauma is associated with AM and EFT. For instance, individuals who experienced childhood sexual abuse exhibited significantly lower AM specificity compared with those not experiencing childhood trauma (Raymaekers et al. 2010), and childhood physical abuse was associated with overgeneralized (less specific) AM in individuals with major depressive disorder (Griffith et al. 2016). A recent review including 48 relevant studies suggested that most of the studies have shown a negative correlation between interpersonal childhood trauma (physical/emotional/sexual abuse, physical/emotional neglect) and AM specificity, though a small number of studies did not find such associations (Borrelli et al. 2024). Only a few studies have examined the association between childhood trauma and EFT. Major depressive disorder patients with childhood trauma produced significantly fewer episodic details with neutral cue words during EFT than healthy controls (HC) (Parlar et al. 2016). In addition, adolescents with childhood trauma imagined fewer specific future events than typically developing participants (Lau‐Zhu et al. 2024).

Childhood trauma is also closely related to schizophrenia. Compared with the general population (Matheson et al. 2012), schizophrenia patients have a higher rate of childhood trauma, which is a risk factor for psychosis (Álvarez et al. 2014; Morgan and Gayer‐Anderson 2016; Varese et al. 2012). Childhood trauma is also associated with clinical symptoms of schizophrenia (Fan et al. 2022; Heins et al. 2011; Neumann et al. 2023). Furthermore, childhood trauma could affect cognitive functioning in schizophrenia patients, such as attention, problem‐solving skills, working memory, and so on (De‐Nardin et al. 2022).

Few studies have examined the relationship between childhood trauma and AM in patients with schizophrenia. A study revealed that specific AM were significantly reduced in both schizophrenia with and without childhood trauma groups compared with HC, and the two patient groups did not show a significant difference (Barry et al. 2021). It was suggested that individuals who have experienced childhood trauma were more likely to generate overgeneral (less specific) AM, which may be associated with functional avoidance (individuals tend not to retrieve specific aversive events to maintain a better emotional state) and neural changes caused by childhood trauma (Brien et al. 2021; Williams et al. 2007). However, no studies have examined the association between childhood trauma and EFT in schizophrenia patients.

There are many similarities between AM and EFT. The constructive episodic simulation hypothesis proposed that EFT was based on flexible recombining and reorganizing the elements retrieved from AM (Schacter and Addis 2007). AM and EFT also share a similar knowledge structure, with abstract and general events stored in higher levels, and specific and episodic events stored in lower levels. People usually search and retrieve events from higher to lower levels; if they stop searching prematurely, they tend to generate more abstract and general events (Cole and Kvavilashvili 2019). Furthermore, AM and EFT have a large overlap of neural basis including the medial prefrontal cortex, posterior cingulate gyrus, and medial temporal lobe (Botzung et al. 2008; Schacter et al. 2012). However, differences were also found between AM and EFT. For example, EFT events showed less strong phenomenological characteristics such as vividness, specificity, and sense of experience than AM since EFT events did not happen yet, while AM events had happened (Anderson and Dewhurst 2009; Berntsen and Bohn 2010; Schubert et al. 2020). But EFT events were more positive than AM events, indicating a positive bias for the future (Rasmussen and Berntsen 2014). Moreover, EFT was more severely impaired in schizophrenia compared with AM (D'Argembeau and Raffard 2008). In addition, the right hippocampus and right frontal cortex activated more strongly during EFT compared with AM, indicating additional psychological processes (Addis et al. 2007; Szpunar et al. 2007; Viard et al. 2011).

Taken together, both AM and EFT have been found to be impaired in patients with schizophrenia. However, the effect of childhood trauma on AM and particularly on EFT in schizophrenia patients remained unclear. The present study aimed to examine the effect of childhood trauma on AM and EFT in schizophrenia patients; we also aimed to examine whether there would be differential impairments between AM and EFT. Based on previous findings, we made the following hypotheses. First, both schizophrenia patients with or without childhood trauma would show AM and EFT impairments compared with HC. Second, patients with childhood trauma would show severer AM and EFT impairments compared with patients without childhood trauma. Third, EFT would have lower specificity and number of details, but higher positive emotions than AM.

## 2 Methods

### 2.1 Participants

Sixty inpatients with schizophrenia (SCZ) were recruited from Zunyi Psychiatric Hospital. They were divided into a childhood trauma group (SCZ + CT, *N* = 41) and a without childhood trauma group (SCZ − CT, *N* = 19) according to the Childhood Trauma Questionnaire (CTQ) with criteria described in the following section. The inclusion criteria of patients were as follows: met the diagnostic criteria for schizophrenia according to the Diagnostic and Statistical Manual of Mental Disorders‐fifth edition (American Psychiatric Association 2013); aged between 18 and 60 years no less than 70 on Intelligence Quotient (IQ); cooperative and able to complete the study. Exclusion criteria were as follows: a history of neurological disorders; a history of brain injury; a history of alcohol/drug dependence/abuse; received electroconvulsive therapy within the past 3 months.

Forty healthy controls (HC) were recruited from the local community via advertisements and completed the study. Participants need to fulfill the following inclusion criteria: no current diagnosis or history of psychiatric disease screened by a trained researcher using the Mini‐International Neuropsychiatric Interview (Sheehan et al. 1998; Si et al. 2009); aged between 18 and 60 years no less than 70 on IQ; cooperative and able to complete the study. Exclusion criteria were as follows: a family history of psychiatric disease, neurological disease; a history of alcohol/drug dependence/abuse; a history of brain injury. About matching criteria, HC were individually matched to patients by age (±5 years) and years of education (±3 years) while keeping gender ratio, mean age, and education years non‐significantly differed among groups.

The present study was approved by the Ethics Committee of Capital Normal University and Zunyi Psychiatric Hospital, and the research was completed in accordance with the Helsinki Declaration. All participants gave written informed consent before the study commenced.

### 2.2 Measures

#### 2.2.1 Childhood Trauma

The Chinese version of the CTQ (Bernstein et al. 2003; Zhao et al. 2005) was adopted to measure childhood trauma. The CTQ is a 28‐item self‐report questionnaire and measures five types of childhood trauma, including sexual abuse, physical abuse, emotional abuse, physical neglect, and emotional neglect. Participants rate each item on a 5‐point scale (1 = *never true*, 2 = *rarely true*, 3 = *sometimes true*, 4 = *often true*, 5 = *very often true*). A higher score means severer trauma. The cutoff for having childhood trauma in each dimension was as follows: sexual abuse ≥ 8, physical abuse ≥ 10, emotional abuse ≥ 13, physical neglect ≥ 10, and emotional neglect ≥ 15. A person is considered to have childhood trauma if any dimension meets the criteria. The Cronbach's alpha was 0.868 for the whole scale in this study.

#### 2.2.2 Remembering the Past and Imagining the Future

**FIGURE 1:**
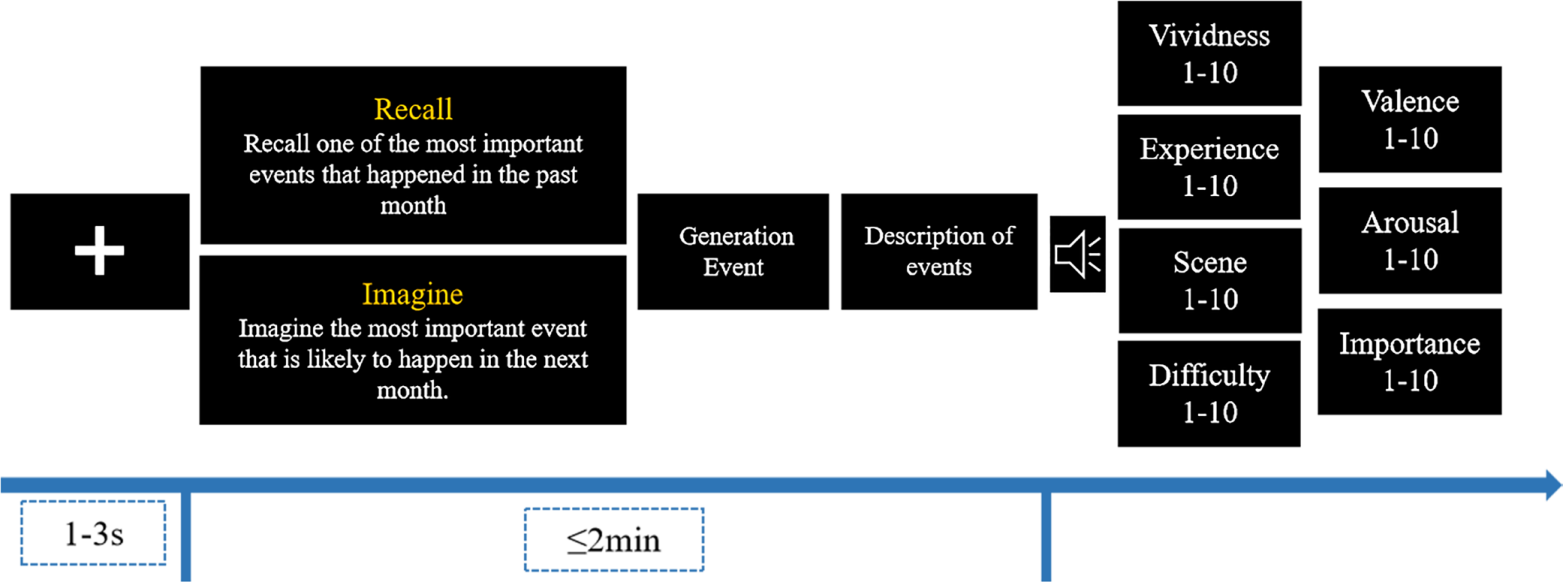
Procedure of one trial of the autobiographical interview task.

The Autobiographical Interview (AI) task was adapted from Levine et al. (2002). The procedure of a trial is shown in Figure 1.

Participants completed two blocks of task (recall and imagine), each block comprised four trials (recall the most important event that happened “within the past month,” “within the past year,” “within the past 5 years,” or “before the past 5 years”; imagine the most important event that might happen “within the next month,” “within the next year,” “within the next 5 years,” or “after the next 5 years”). The order of blocks was counterbalanced between participants. The 4 trials within each block were presented in a fixed order (from recent to distant).

Each trial began with a fixation for a random duration between 1 and 3 s (on average 2 s), then the task cue appeared. The cue included two lines: The first line indicates task condition (“recall” or “imagine”); the second line was the cue. The cue slide was present on the screen during the event generation and description phase. The duration from the appearance of the cue to the completion of description was no more than 2 min. If 2 min passed and participants were still describing, a “beep” was played and the program would automatically skip to the next slide. If the participants finished the description ahead of time, they could press the space key, then a “beep” was played and the program would skip to the next slide. After the description of each event, participants rated the event on a scale of 1–10 for the following aspects: vividness (1 = *not vivid at all*, 10 = *very vivid*), sense of experience (1 = *not strong at all*, 10 = *very strong*), scene details (1 = *not rich at all*, 10 = *very rich*), difficulty in generation (1 = *very difficult*, 10 = *not difficult at all*), valence (1 = *very negative*, 10 = *very positive*), arousal (1 = *not strong at all*, 10 = *very strong*) and importance (1 = *not important at all*, 10 = *very important*). Participants' descriptions were audio recorded; the audio recordings were transcribed into text for rating after the experiment was completed. Ratings included the amount of internal and external detail, specificity, richness of time/place, perception, thought/emotion for each event. A detailed description of the indexes and scoring criteria is provided in the Supporting Information S1. Two raters rated 20% of the data independently, and there was good inter‐rater reliability for all indexes (ICC ≥ 0.796). Then a rater blind to participant groups rated all data.

#### 2.2.3 Clinical Symptoms

The Positive and Negative Syndrome Scale (PANSS) (Kay et al. 1987; Si et al. 2004) was used to measure the severity of clinical symptoms of patients. All the patients were rated by the same psychiatrist through interview.

#### 2.2.4 IQ

The Wechsler adult intelligence scale‐Chinese version (Gong 1992) was used to measure the IQ of participants.

### 2.3 Statistical Analysis

All data were analyzed via SPSS (Version 25.0). One‐way ANOVA and chi‐square test were performed to compare group differences in demographics. Independent sample *t*‐tests were performed to compare clinical variables between the two patient groups. A series of 3 (Group: SCZ + CT, SCZ − CT, HC) × 2 (Time Direction: past, future) repeated measures ANOVAs were conducted on the measures rated by participants and rated by raters in the remember and imagine task. If the main effect of Group was significant, further pairwise comparisons with Bonferroni correction was conducted; if the interaction was significant, simple effect analysis was conducted.

## 3 Results

### 3.1 Group Comparisons on Demographics and Clinical Information

The three groups were matched on gender, age, and years of education (all *p* values > 0.050), but had significant differences on IQ (*F*(2,97) = 10.02, *p* < 0.001), with both patient groups having lower IQ than HC. The two patient groups were matched on clinical variables. See Table 1 for details.

**TABLE 1:** Group comparisons on demographics and clinical information.

	SCZ + CT (*N* = 41)		SCZ − CT (*N* = 19)		HC (*N* = 40)		*χ* ^2^ */F/t*	*p*
	Mean	SD	Mean	SD	Mean	SD		
Gender (males:females)	35:6		12:7		31:9		3.74	0.154
Age (years)	39.56	9.07	37.89	11.28	36.83	12.67	0.63	0.536
Years of education	9.83	3.73	10.26	2.60	10.80	2.51	1.00	0.370
IQ	94.68	15.41	95.47	14.83	108.05	12.98	10.02	< 0.001
Duration of illness (years)	12.93	7.35	11.42	7.93	—	—	−0.72	0.475
Medication (CPZ mg/d)	149.78	106.46	173.74	142.10	—	—	0.73	0.470
PANSS_total	60.97	10.59	56.84	11.66	—	—	−1.35	0.183
PANSS_Positive	9.13	5.27	8.84	3.99	—	—	−0.21	0.835
PANSS_Negative	23.05	6.99	20.63	7.80	—	—	−1.19	0.239
PANSS_General	28.79	4.01	27.37	4.68	—	—	−1.20	0.234

*Note*: PANSS data were missing for two patients with childhood trauma.

Abbreviations: CPZ, chlorpromazine equivalence; HC, healthy controls; PANSS, Positive and Negative Syndrome Scale; SCZ + CT, schizophrenia with childhood trauma; SCZ − CT, schizophrenia without childhood trauma.

### 3.2 Analyses on the Indexes for the Autobiographical Interview Task

#### 3.2.1 Indexes of Raters

**FIGURE 2:**
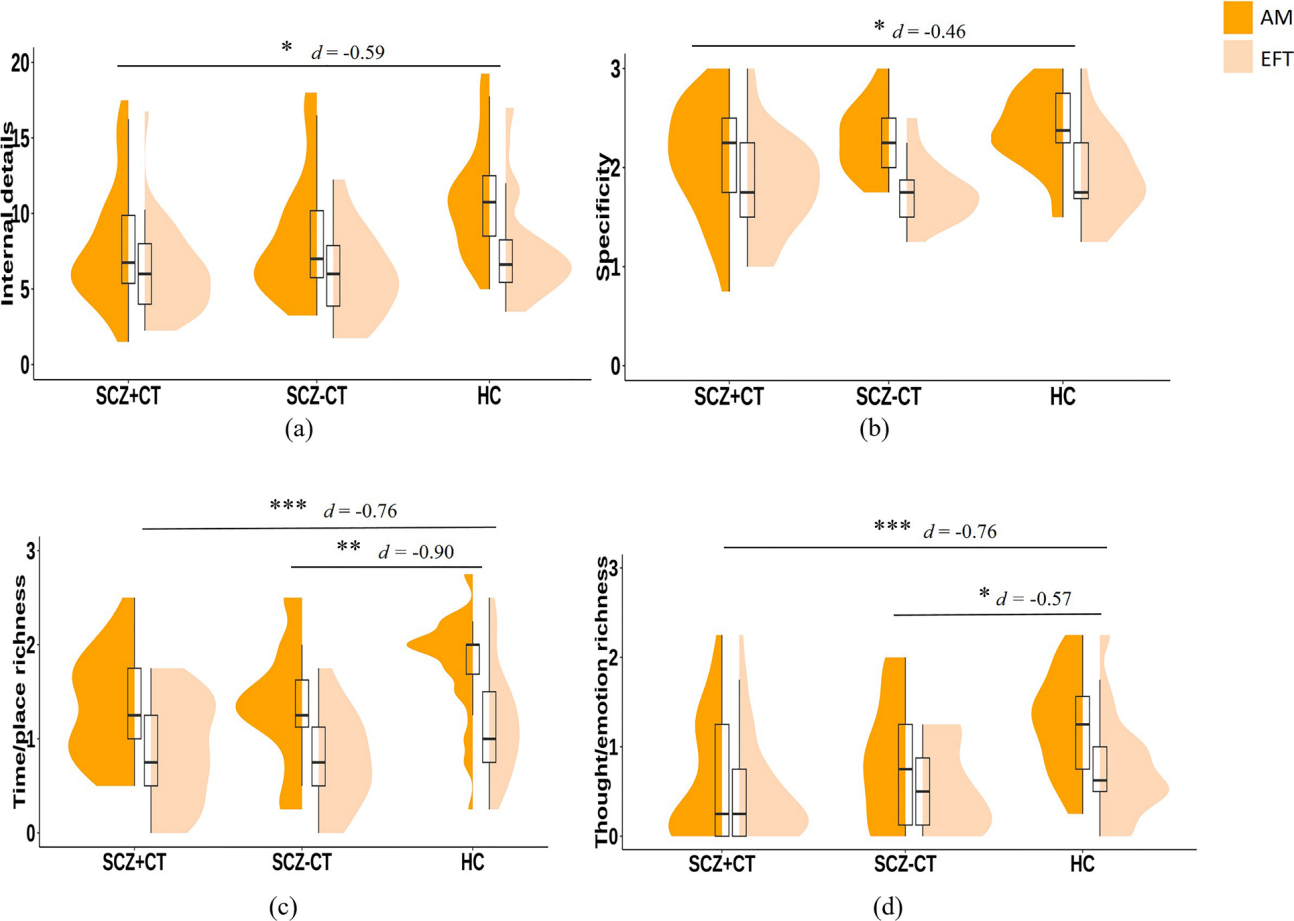
Group comparisons on internal details (a), specificity (b), time/place richness (c), thought/emotion richness (d). AM, autobiographical memory; EFT, episodic future thinking; HC, healthy control; SCZ + CT, schizophrenia with childhood trauma; SCZ − CT, schizophrenia without childhood trauma. *p < 0.05; **p < 0.01; ***p < 0.001.

The main effect of Group was significant on the number of internal details (*F*(2,97) = 5.04, *p* = 0.008, *η*_*p*_^2^ = 0.09, 95% CI [0.007, 0.203]), specificity (*F*(2,97) = 3.20, *p* = 0.045, *η*_*p*_^2^ = 0.06, 95% CI [0, 0.160]), time/place richness (*F*(2,97) = 10.34, *p* < 0.001, *η*_*p*_^2^ = 0.18, 95% CI [0.051, 0.298]) and thought/emotion richness (*F*(2,97) = 8.87, *p* < 0.001, *η*_*p*_^2^ = 0.16, 95% CI [0.038, 0.275]). Pairwise comparisons revealed that SCZ + CT generated significantly fewer internal details (*p* = 0.010) and lower specificity (*p* = 0.042) than HC. The SCZ + CT (*p* < 0.001) and SCZ − CT (*p* = 0.002) groups had lower time/place richness than HC. The SCZ + CT (*p* < 0.001) and SCZ − CT (*p* = 0.047) groups had lower thought/emotion richness than HC. There were no significant differences between SCZ + CT and SCZ − CT on any of these indexes (see Figure 2 and Table 2).

**TABLE 2:** Comparison of rater‐rated indexes.

	SCZ + CT (*N* = 41)				SCZ − CT (*N* = 19)				HC (*N* = 40)				Group		Time direction		Interaction	
	AM		EFT		AM		EFT		AM		EFT							
	Mean	SD	Mean	SD	Mean	SD	Mean	SD	Mean	SD	Mean	SD	*F*	*p*	*F*	*p*	*F*	*p*
Internal details	8.57	5.11	6.06	2.74	9.34	5.76	5.88	2.84	11.85	4.48	7.54	3.25	5.04	0.008	59.21	< 0.001	1.89	0.157
External details	3.01	2.48	2.65	1.91	3.91	2.54	4.22	2.90	3.73	2.66	3.14	2.29	2.46	0.091	0.59	0.446	0.80	0.452
Specificity	2.09	0.54	1.82	0.47	2.29	0.35	1.75	0.30	2.39	0.38	1.91	0.42	3.20	0.045	61.93	< 0.001	2.45	0.092
Time/place	1.33	0.55	0.87	0.54	1.36	0.64	0.78	0.49	1.82	0.52	1.18	0.55	10.34	< 0.001	67.12	< 0.001	0.87	0.422
Perception	0.79	0.68	0.41	0.52	0.74	0.53	0.39	0.47	0.86	0.77	0.32	0.52	0.03	0.966	27.59	< 0.001	0.71	0.493
Thought/emotion	0.64	0.67	0.49	0.56	0.83	0.70	0.53	0.48	1.28	0.54	0.74	0.56	8.87	< 0.001	21.20	< 0.001	3.40	0.038

Abbreviations: AM, autobiographical memory; EFT, episodic future thinking; HC, healthy control; SCZ + CT, schizophrenia with childhood trauma; SCZ − CT, schizophrenia without childhood trauma.

For the main effect of Time Direction, except for external details, all other indexes were significantly higher in AM than EFT (see Table 2). The interaction between Group and Time Direction was significant on thought/emotion richness (*F*(2,97) = 3.40, *p* = 0.038, *η*_*p*_^2^ = 0.07, 95% CI [0, 0.165]). Simple effect analyses revealed that emotional/thought richness of AM was significantly lower in SCZ + CT (*p* < 0.001) and SCZ − CT (*p =* 0.037) than the HC; there was no significant difference in emotional/thought richness of EFT among the three groups. Other interactions were non‐significant.

#### 3.2.2 Participants Self‐Rating Indexes

As shown in Table 3, the main effect of Group was not significant on any index (all *p* values > 0.050). The main effect of Time Direction was significant on all indexes (*p* values < 0.050) except for arousal (*p* = 0.908). EFT had higher valence and importance than AM (*p* < 0.001), whereas the other indexes (vividness, sense of experience, scene details, difficulty, all *p* values < 0.001) showed a reversed pattern.

**TABLE 3:** Comparisons of participants self‐rating indexes.

	SCZ + CT (*N* = 41)				SCZ − CT (*N* = 19)				HC (*N* = 40)				Group		Time direction		Interaction	
	AM		EFT		AM		EFT		AM		EFT							
	Mean	SD	Mean	SD	Mean	SD	Mean	SD	Mean	SD	Mean	SD	*F*	*p*	*F*	*p*	*F*	*p*
Vividness	7.57	1.47	7.21	1.59	7.68	1.48	7.38	1.52	7.66	1.37	6.94	1.52	0.17	0.840	18.21	< 0.001	1.68	0.191
Sense of experience	7.89	1.54	7.36	1.40	7.46	1.49	7.24	2.02	8.19	1.26	7.14	1.22	0.40	0.671	21.76	< 0.001	3.57	0.032
Scene details	7.05	1.49	6.62	1.49	6.93	1.37	6.74	1.35	7.36	1.40	6.54	1.57	0.09	0.915	19.23	< 0.001	2.67	0.075
Difficulty	7.10	1.55	6.77	1.48	7.36	1.48	6.54	1.60	7.95	1.19	7.03	1.21	2.26	0.110	28.63	< 0.001	2.61	0.078
Valence	6.32	1.65	6.99	1.53	6.03	1.59	7.21	1.56	6.08	1.42	6.79	1.27	0.31	0.735	31.70	< 0.001	0.96	0.387
Arousal	6.57	1.82	6.40	2.02	6.80	1.57	6.99	1.27	6.34	1.20	6.37	1.16	0.87	0.424	0.01	0.908	0.83	0.441
Importance	7.14	1.89	7.38	1.80	7.80	1.16	8.39	1.22	7.39	1.64	7.81	1.29	2.22	0.114	8.61	0.004	0.49	0.613

Abbreviations: AM, autobiographical memory; EFT, episodic future thinking; HC, healthy control; SCZ + CT, schizophrenia with childhood trauma; SCZ − CT, schizophrenia without childhood trauma.

The interaction between Group and Time Direction was significant on sense of experience (*F*(2,97) = 3.57, *p* = 0.*032*, *η*_*p*_^2^ = 0.07, 95% CI [0, 0.170]); both the SCZ + CT (*p* = 0.006) and the HC (*p* < 0.001) groups showed higher sense of experience in AM than in EFT. In contrast, no significant difference between AM and EFT was found in the SCZ − CT group (*p* = 0.424). The interaction between Group and Time Direction was not significant on other indexes.

## 4 Discussion

The present study revealed that both SCZ + CT and SCZ − CT had deficits in AM and EFT, but no significant differences were found between the two patient groups. In addition, AM and EFT differed in phenomenological characteristics.

SCZ + CT generated events with fewer internal details, lower specificity, time/place richness, and emotion/thought richness during AM and EFT than HC, and SCZ − CT generated events with lower time/place richness and emotion/thought richness than HC. These results are partially consistent with our hypothesis and consistent with previous studies on AM and EFT in schizophrenia (Chen et al. 2016; Kwok et al. 2021; Zhang et al. 2019). SCZ − CT exhibited lower time/place richness and emotion/thought richness during AM and EFT than HC, suggesting these impairments are related to schizophrenia, not to childhood trauma. Schizophrenia is a disorder with a temporal dimension of self‐impairment, which manifests in a sense of subjective temporal disconnection and difficulty in relating the present to the past and the future (Northoff et al. 2021). This disorder not only affects their ability to project themselves to different times but also makes it difficult to retrieve specific details from memory (D'Argembeau and Raffard 2008), which leads to deficits in AM and EFT. Schizophrenia patients also suffer from executive dysfunction (Pietrzykowski et al. 2022), and retrieving memories with details requires executive resources. Therefore, patients with schizophrenia have difficulties in generating AM and EFT with details (Williams et al. 2007). Moreover, schizophrenia patients have deficits in scene construction (i.e., the process by which the mind produces and maintains a complex and coherent scene), which is a core component of AM and EFT (Raffard et al. 2010). SCZ − CT were also lower in the amount of internal details (*d*_AM_ = −0.49, *d*_EFT_ = −0.54) and specificity (*d*_AM_ = −0.27, *d*_EFT_ = −0.44) than HC, but these differences did not reach significance. It may be due to the fact that most of the previous studies did not differentiate schizophrenia patients into those with or without childhood trauma (Berna et al. 2016; Hallford et al. 2018); the results that schizophrenia patients exhibited deficits in AM and EFT specificity might be confounded by the effect of childhood trauma.

The significance level of deficits in time/place richness and emotion/thought richness of AM and EFT was higher in SCZ + CT than that of SCZ − CT compared with HC, and SCZ + CT showed a significant difference on more indexes compared with HC than SCZ − CT compared with HC. However, there was no significant difference for the direct comparison between SCZ + CT and SCZ − CT. Therefore, childhood trauma may have a subtle effect on AM and EFT deficits in schizophrenia patients. SCZ + CT had a wider AM and EFT deficits than SCZ − CT compared with HC. This might be that childhood trauma exacerbates cognitive deficits in schizophrenia (Fang et al. 2020; Wang et al. 2021), which may lead to a decline in AM and EFT. Another possibility is that according to the Capture and Rumination‐Functional Avoidance‐impaired Executive control (CaR‐FA‐X) model (Williams et al. 2007), childhood trauma may cause schizophrenia patients to stop searching from the autobiographical knowledge base before retrieving specific events as a way to avoid experiencing more negative emotions. The CaR‐FA‐X model provides a useful framework for understanding the observed deficits in EFT among patient groups (Ricarte et al. 2017), particularly the Functional Avoidance part. Lead schizophrenia patients with childhood trauma may engage in avoidance strategies to prevent them from imagining future negative events. This avoidance may lead to the construction of overgeneral and emotionally flat future events, which lack the rich details and experiential quality observed in HC. Therefore, SCZ + CT exhibited less specific and detailed AM and EFT. The difference between SCZ + CT and SCZ − CT may become significant with a larger sample size.

As to the difference between EFT and AM, EFT was more positive and important than AM events, while AM was easier to generate, more vivid, more detailed, and had more sense of experience than EFT events, which were consistent with previous studies (Berntsen and Bohn 2010; Rasmussen and Berntsen 2012). According to the constructive episodic simulation hypothesis, we flexibly recombine and reorganize elements from the past to imagine future events (Schacter and Addis 2007), suggesting that EFT requires more constructive efforts than AM (Addis et al. 2009). Therefore, AM was easier to generate than EFT, and AM events might be more vivid, more detailed, and had more sense of experience than EFT events. In terms of emotional valence, people had more positive emotions when imagining future events than remembering past events, demonstrating that people have a positive bias toward the future and a positive expectation for the future (Lench and Bench 2012; Taylor and Brown 1988; Yue et al. 2021). Schizophrenia patients also have a positive bias toward the future (Raffard et al. 2015). The CaR‐FA‐X model may be used to explain this bias, as patients might construct positive future scenarios to avoid negative emotions or psychological distress (Williams et al. 2007). A meta‐analysis on studies conducted in the general population also showed that remembered past events were more vivid than imagined future events (*g* = −0.61), and imagined future events were associated with more positive emotions (*g* = 0.56) (Morton and MacLeod 2023). Therefore, although AM and EFT are closely related, there are also differences between them.

For the interaction between Group and Time Direction, in terms of emotional/thought richness, both patient groups showed deficits in AM but not in EFT. Patients with schizophrenia typically exhibit negative symptoms such as apathy and show increased negative emotions, which makes it difficult for them or reluctant to recall the emotional/thought details associated with past events. For EFT, it might be because HC also had difficulty imagining the future, which might be associated with their low level of education. When they imagine the future, they tend to pay more attention to entity details such as time and place and pay less attention to details such as emotions and inner feelings and thoughts. For the sense of experience, SCZ + CT and HC scored higher in AM than in EFT, because AM events had already happened and EFT events had not yet occurred. SCZ − CT also showed a similar trend but did not reach significance, which might be due to the small sample size in the SCZ − CT group.

There are several limitations in this study. First, the sample size of the SCZ − CT group was small; further studies should recruit a larger sample size. A post hoc power analysis was conducted using G*Power v3.1.9.2 (Faul et al. 2007) to assess the achieved statistical power for detecting the Group × Time Direction interaction in ANOVA. Based on the obtained partial eta squared values ranging from 0.01 to 0.069 in the present study (corresponding to Cohen's *f* values between 0.10 and 0.27), the estimated statistical power ranged from 0.408 to 0.999, suggesting the present study was underpowered to detect some of the effects. Second, this study did not analyze different dimensions of childhood trauma. The five dimensions of childhood trauma may have differential effects on AM and EFT; future studies may examine this issue. Third, this study only used participants' self‐reported childhood trauma measure; future studies may use objectively documented childhood trauma measures, since it was suggested that objectively recorded childhood trauma had a larger impact on cognition than self‐reported childhood trauma (Danese and Widom 2024). Finally, we did not distinguish HC into those with or without childhood trauma, as previous studies have also not distinguished between them (Aas et al. 2019).

Notwithstanding the above limitations, this study deepened our understanding of the psychopathological mechanism of schizophrenia. Examining the effect of childhood trauma on AM and EFT in schizophrenia can also provide guidance for disease treatment. When clinical workers are developing intervention plans for schizophrenia patients, they may take childhood trauma into consideration and incorporate relevant strategies.

## 5 Conclusions

Both SCZ + CT and SCZ − CT groups exhibited AM and EFT impairments; the SCZ + CT group had wider impairments than the SCZ − CT group compared with HC. However, the direct comparison between SCZ + CT and SCZ − CT did not show a significant difference. These results suggest that childhood trauma had a subtle effect on AM and EFT impairments in schizophrenia patients.

## Ethics Statement

Approval was obtained from the Ethics Committees of Capital Normal University and Zunyi Psychiatric Hospital, and the research was completed in accordance with the Helsinki Declaration.

## Conflicts of Interest

The authors declare no conflicts of interest.

## Supporting information


**Data S1:** Supporting Information.

## Data Availability

The data that support the findings of this study are available from the corresponding author upon reasonable request.
