# Supplementary material for: Effects of Childhood Trauma on Remembering the Past and Imagining the Future in Schizophrenia
Source: Psych J. 2025 Jul 31;14(6):867–76. doi: 10.1002/pchj.70044 (PMC12702601; doi:10.1002/pchj.70044)
Supplement: Supplementary file 1 — Data S1: Supporting Information. [file PCHJ-14-867-s001.docx]

**Supplementary materials**

**Rater rating indexes and scoring criteria**

*Internal details*

Internal details are details related to the main event described, which includes detailed words such as event, time/place, perceptions, emotion/thought, and so on. The amount of internal detail is calculated by counting the number of relevant details.

*External details*

External details are details that are not related to the main event being described, including detailed words about unrelated events, time/place, perceptions, emotion/thought, repetitive details, and other semantic details. The amount of external details is calculated by counting the number of irrelevant details.

*Specificity*

Events are categorized into four types based on specificity and rated on a scale of 0-3 according to the criteria below.

3 points: Specific event, the event occur at a specific time and place and lasted no more than one day

2 points: Extended event, events occur over a period of time lasting more than one day

1 point: Category event, generalizations about a category of events that may happen repetitively

0 points: No events were mentioned

*Detail richness*

The richness of three types of details, i.e., time/place, perception, and emotion/thought details, are rated on a scale of 0-3 according to the following criteria.

3 points: Very rich in detail, highly specific and vividly described

2 points: Detailed descriptions are richer and more specific

1 point: Lack of specificity in details and broad generalizations in descriptions

0 points: No mention of the relevant words of detail

**Table S1 Effect Sizes for Rater-Rated Indexes Between SCZ+CT and SCZ-CT Groups**

|  | SCZ+CT (*N*=41) | | | | | SCZ-CT (*N*=19) | | | |  |  |
| --- | --- | --- | --- | --- | --- | --- | --- | --- | --- | --- | --- |
|  | AM | | | EFT | | AM | | EFT | | Effect Sizes | |
|  | *Mean* | | *SD* | *Mean* | *SD* | *Mean* | *SD* | *Mean* | *SD* | Cohen’s *d* _AM_ | Cohen’s *d* _EFT_ |
| Internal details | 8.57 | 5.11 | | 6.06 | 2.74 | 9.34 | 5.76 | 5.88 | 2.84 | -0.14 | 0.06 |
| External details | 3.01 | 2.48 | | 2.65 | 1.91 | 3.91 | 2.54 | 4.22 | 2.90 | -0.36 | -0.64 |
| Specificity | 2.09 | 0.54 | | 1.82 | 0.47 | 2.29 | 0.35 | 1.75 | 0.30 | -0.44 | 0.18 |
| Time/place | 1.33 | 0.55 | | 0.87 | 0.54 | 1.36 | 0.64 | 0.78 | 0.49 | -0.05 | 0.17 |
| Perception | 0.79 | 0.68 | | 0.41 | 0.52 | 0.74 | 0.53 | 0.39 | 0.47 | 0.08 | 0.04 |
| Thought/emotion | 0.64 | 0.67 | | 0.49 | 0.56 | 0.83 | 0.70 | 0.53 | 0.48 | -0.28 | -0.08 |

*Note*. SCZ+CT = Schizophrenia with childhood trauma; SCZ-CT = Schizophrenia without childhood trauma; AM = autobiographical memory; EFT = episodic future thinking.

****Table S2** Effect Sizes for Participants Self-rating Indexes Between SCZ+CT and SCZ-CT Groups**

|  | SCZ+CT (*N*=41) | | | | | SCZ-CT (*N*=19) | | | |  |  |
| --- | --- | --- | --- | --- | --- | --- | --- | --- | --- | --- | --- |
|  | AM | | | EFT | | AM | | EFT | | Effect Sizes | |
|  | *Mean* | | *SD* | *Mean* | *SD* | *Mean* | *SD* | *Mean* | *SD* | Cohen’s *d* _AM_ | Cohen’s *d* _EFT_ |
| Vividness | 7.57 | 1.47 | | 7.21 | 1.59 | 7.68 | 1.48 | 7.38 | 1.52 | -0.07 | -0.11 |
| Sense of experience | 7.89 | 1.54 | | 7.36 | 1.40 | 7.46 | 1.49 | 7.24 | 2.02 | 0.28 | 0.07 |
| Scene details | 7.05 | 1.49 | | 6.62 | 1.49 | 6.93 | 1.37 | 6.74 | 1.35 | 0.08 | -0.08 |
| Difficulty | 7.10 | 1.55 | | 6.77 | 1.48 | 7.36 | 1.48 | 6.54 | 1.60 | -0.17 | 0.15 |
| Valence | 6.32 | 1.65 | | 6.99 | 1.53 | 6.03 | 1.59 | 7.21 | 1.56 | 0.18 | -0.14 |
| Arousal | 6.57 | 1.82 | | 6.40 | 2.02 | 6.80 | 1.57 | 6.99 | 1.27 | -0.14 | -0.35 |
| Importance | 7.14 | 1.89 | | 7.38 | 1.80 | 7.80 | 1.16 | 8.39 | 1.22 | -0.42 | -0.66 |

*Note*: SCZ+CT = Schizophrenia with childhood trauma; SCZ-CT = Schizophrenia without childhood trauma; AM = autobiographical memory; EFT = episodic future thinking
